# Prevalence of hypertension and determinants of treatment-seeking behaviour among adolescents and young adults in India: an analysis of NFHS-4

**DOI:** 10.1093/pubmed/fdac006

**Published:** 2022-03-01

**Authors:** Yuvaraj Krishnamoorthy, Sathish Rajaa, Sudheera Sulgante, Palanivel Chinnakali, Nidhi Jaswal, Sonu Goel

**Affiliations:** Department of Community Medicine, ESIC Medical College and PGIMSR, Chennai, Tamil Nadu 600078, India; Department of Community Medicine, ESIC Medical College and PGIMSR, Chennai, Tamil Nadu 600078, India; Department of Community Medicine, Bidar Institute of Medical Sciences, Bidar, Karnataka 585401, India; Department of Preventive and Social Medicine, Jawaharlal Institute of Postgraduate Medical Education and Research, Puducherry 160012, India; Department of Community Medicine and School of Public Health, Post Graduate Institute of Medical Education and Research, Chandigarh 160012, India; Department of Community Medicine and School of Public Health, Post Graduate Institute of Medical Education and Research, Chandigarh 160012, India; School of Medicine and Health Research Institute (HRI), University of Limerick V94T9PX, Ireland; Department of Human and Health Sciences, Sketty, Swansea University, SA 2 8PP, United Kingdom

**Keywords:** adolescents, hypertension, India, NFHS, young adult

## Abstract

**Background:**

Previous evidences have reported that almost three-fourth of young hypertensives are not seeking care for their condition leading to severe complications. This study was conducted to assess the determinants of treatment-seeking behaviour among the young hypertensives in India.

**Methods:**

The National Family Health Survey-4 data were analysed. Sampling weights and clustering was accounted using *svyset* command. Screening, awareness, prevalence and control status were reported with 95% confidence interval (CI). Poisson regression was done to identify the determinants of treatment-seeking behaviour.

**Results:**

In total, 13.8% of younger adults had hypertension, 51.1% were aware of their status and 19.5% sought treatment. Participants in 15–19 years (adjusted Prevalence Ratio (aPR) = 0.70) and 20–29 years (aPR = 0.63), male gender (aPR = 0.84), Muslim religion (aPR = 1.14), urban region (aPR = 0.87), secondary (aPR = 0.88) and higher education (aPR = 0.86), residing in Northern (aPR = 0.79), Central (aPR = 0.76), Southern region (aPR = 0.65), preferring home treatment, medical shop or any other care (aPR = 0.63) were significant determinants of treatment-seeking behaviour.

**Conclusion:**

More than 1 in 10 younger adults in India have hypertension and only half of them were aware of their status and one-fifth sought treatment. Adolescents, males, Hindus, urban population, higher education and residing in Northern, Central and Southern region had poor treatment-seeking behaviour.

## Introduction

Non-communicable diseases (NCDs), also known as lifestyle diseases, have become one of the leading causes of deaths worldwide.^1^ Almost 60% of all the deaths and 50% of all the morbidity burden is contributed by the major NCDs globally.^2^ Amongst them, hypertension (HTN) has emerged as an important public health concern. Owing to the lack of recognizable signs and symptoms in hypertension, most of the times people remain unaware of this condition. Undiagnosed and untreated HTN for longer time can lead to severe form of disease leading to serious complications and mortality. WHO has rated HTN as one of the major causes of premature mortality worldwide.^3^ Henceforth, consistency in screening for early diagnosis and adequate management with life style modification is needed for effective reduction in the prevalence of high blood pressure (BP).

Several strategies have been developed throughout the world to lower the hypertension burden. Government of India has also introduced several initiatives such as ‘National programme for prevention and control of cancer, diabetes, cardiovascular diseases and stroke (NPCDCS)’, population level screening of hypertension and ‘India Hypertension Control Initiative (IHCI)’ to decrease the deaths and disability.^4^^,^^5^ Most of these strategies are focussed on middle aged and older age group people. However, there is an increasing trend of hypertension among the people belonging to younger age group over the past decade.^6^

Young hypertension can be defined as the hypertension occurring in people <40 years of age.^7^ Almost one-third of young hypertension is caused by some underlying secondary causes.^8^ Though secondary hypertension can be cured when compared with the essential hypertension, which usually lasts lifelong, secondary hypertension causes severe end organ damage more than the essential hypertension.^8^ Hence, a holistic approach to evaluate the younger population for suspected hypertension is important.

Another important dimension that requires focus is the awareness about one’s own hypertension status and treatment-seeking behaviour among the younger population. Since hypertension is considered an iceberg disease, all the adults aged ≥ 18 years should be screened for raised BP. However, previous evidences from India showed that only half of the people with hypertension knew their hypertension status.^9^ Even after diagnosis, almost three-fourth are not seeking care from health facility leading to severe complications and end organ damage.^9^ Even though, several studies were conducted around India to determine the burden of young hypertension,^10–12^ only few studies tried to explore the treatment-seeking behaviour of the young hypertension^13^ and there was no large-scale survey assessing the care-seeking behaviour among young hypertensives. National Family Health Survey-4 (NFHS-4) data provide sufficient opportunity to study about the prevalence of hypertension among adolescents and younger adults in India.^14^ In addition to the point estimates, understanding the treatment-seeking behaviour and its determinants might help in devising new strategies to increase the treatment coverage rate. Hence, this study was conducted as a secondary data analysis of NFHS-4 data to determine the prevalence of hypertension the determinants of treatment-seeking behaviour among adolescents and young adults in India.

## Methods

### Study design

A secondary data analysis was conducted using nationally representative data of NFHS-4 gathered from the Demographic Health Survey (DHS) programme. NFHS survey has been conducted to capture data on various health indicators the Indian population.

### Study setting

India, the second most populated country in the world, is having a population of roughly 130 crores and is divided into 30 states and 6 union territories (UT). Each state is sub-divided into districts and further districts into census enumeration blocks (CEB)/wards in urban area and villages/taluk in rural area.

In India, opportunistic screening for hypertension should be done for all the individuals aged ≥18 years for the diagnosis of young hypertension.^15^ India was also the first country worldwide to adopt the NCD global monitoring framework and action plan, which included nine targets including the target on reducing the burden of hypertension.^16^

### Participants

Participants < 40 years (15–39 years) were taken as study population for the current study analysis. Variables extracted for these study population consists of the following: independent variables extracted were socio-demographic characteristics such as age, gender, education, wealth index, marital status, type of residence, religion, caste/tribe, geographical region, health insurance coverage and general healthcare seeking behaviour. Dependent variable was the treatment-seeking behaviour among the young hypertensives.

### Data sources

Self-reported case of hypertension was ascertained in the survey based on ‘yes’ or ‘no’ response to the question whether the respondents were told that they have high BP on ≥2 occasions by the physician. Newly diagnosed hypertension was defined by the Joint National Committee (JNC’s) eight hypertension guidelines.^17^ All the participants underwent three BP measurements during the survey. Participants with systolic blood pressure (SBP) reading ≥ 140 mmHg and/or diastolic blood pressure (DBP) reading ≥ 90 mmHg while taking the average of second and third measurements were identified as newly diagnosed cases. For the assessment of treatment-seeking behaviour, question on whether sought treatment and currently on medication was asked among the respondents who were told that they had high BP on ≥2 occasions by a physician.

### Study size

In NFHS-4 survey, two-stage sampling method was used for the selection of villages (in rural areas) and CEBs (in urban areas). The household selection process, data sources, its validation and data collection procedure have been described comprehensively as a separate report.^14^

In total, 699 686 females and 112 122 males have completed the questionnaire. Since the current study focuses on young hypertension, the responses of 631 876 participants (15–39 years) who were interviewed regarding the hypertension status were included into the analysis.

### Statistical methods

We obtained the dataset from the DHS website in.dta format and it was imported into STATA 14.2 (StataCorp, College Station, TX, USA) for analysing the data. Variables necessary for the analysis such as age, gender, education, wealth index, marital status, type of residence, religion, caste/tribe, geographical region, health insurance coverage and general healthcare seeking behaviour were kept in the dataset and all other variables were dropped for ease of analysis. These co-variates were selected after reviewing the previous literature on similar studies, in addition to obtaining the opinion of public health experts in this regard.

Then, sampling weights were adjusted while performing the analysis, to account the differential probabilities of participant selection. Sample design and clustering was also accounted using *svyset* command. Screening, prevalence, awareness and control status of young hypertension (belonging to age group < 40 years) were reported with 95% confidence interval (CI). Univariable and multivariable Poisson regression was performed to identify the determinants of treatment-seeking behaviour among young hypertensives. Unadjusted and adjusted prevalence ratio (PR) with 95%CI was reported. Variables with *P* value < 0.20 in the univariable model were considered into the multivariable regression model. Variables with *P* value < 0.05 in the multivariable model were considered statistically significant determinants of treatment-seeking behaviour.

Poisson regression was performed instead of the commonly employed logistic regression technique as the logistic regression only provides the odds ratio, whereas PR can be estimated by the Poisson regression (better measure for reporting the effect estimate in cross-sectional surveys). In addition, it is difficult to infer the odds ratio for cross-sectional studies as there is confusion between odds or risk, leading to erroneous quantitative interpretation.

## Results

In total, 631 876 participants aged 15–39 years were monitored for hypertension in the NFHS-4 survey. As shown in Table 1, majority of the participants (43.3%) belonged to the age group between 20 and 29 years. Females consisted of 87.5% of the study population. Most of the participants (67%) were currently married; more than three-fourth belonged to Hindu religion; more than two-third had secondary to higher educational qualification; majority (66.6%) were living in rural area. Almost one-fourth of the study participants belonged to Central region. Some form of health insurance scheme covered only one-fourth of the participants; more than half of them generally seek care in private healthcare facilities.

**Table 1:** Socio-demographic characteristics of the study participants assessed for HTN in NFHS-4 survey 2015–16, India, *N* = 631 876

Socio-demographic characteristics	Frequency, *N* (unweighted proportion %)	Weighted proportion (95% CI)
**Age category (in years)**		
15–19	144 211 (22.8)	22.4 (22.3–22.6)
20–29	271 957 (43.0)	43.3 (43.1–43.5)
30–39	215 708 (34.1)	34.3 (34.1–34.5)
**Gender**		
Male	80 046 (12.7)	12.5 (12.3–12.8)
Female	551 830 (87.3)	87.5 (87.2–87.7)
**Marital status (*N* = 631 846)**		
Never married	207 205 (32.8)	30.9 (30.7–31.1)
Currently married	411 407 (65.1)	67.0 (66.7–67.2)
Widowed/separated/divorced	13 234 (2.1)	2.1 (2.0–2.2)
**Religion**		
Hindu	465 383 (73.6)	80.0 (79.4–80.5)
Muslim	90 359 (14.3)	14.5 (14.0–15.0)
Christian	45 808 (7.3)	2.2 (2.1–2.4)
Others^a^	30 326 (4.8)	3.2 (3.1–3.5)
**Education status (*N* = 630 701)**		
No formal education	122 522 (19.5)	19.0 (18.7–19.3)
Primary	79 083 (12.5)	12.5 (12.3–12.6)
Secondary	344 555 (54.6)	53.7 (53.4–54.0)
Higher	84 541 (13.4)	14.8 (14.5–15.1)
**Caste/tribe (*N* = 605 513)**		
Scheduled caste^c^	115 990 (19.2)	21.8 (21.4–22.2)
Scheduled tribe^c^	115 602 (19.1)	9.7 (9.4–10.0)
Other backward class	245 039 (40.5)	44.9 (44.4–45.5)
None of the above	125 788 (20.8)	22.9 (22.4–23.4)
Don’t know	3094 (0.5)	0.7 (0.6–0.8)
**Wealth index**		
Poorest (I quintile)	120 440 (19.1)	18.0 (17.6–18.4)
Poorer (II quintile)	136 768 (21.6)	20.1 (19.8–20.4)
Middle (III quintile)	134 933 (21.4)	21.0 (20.7–21.2)
Richer (IV quintile)	125 323 (19.8)	21.0 (20.7–21.3)
Richest (V quintile)	114 412 (18.1)	19.9 (19.4–20.4)
**Residence**		
Urban	182 812 (28.9)	33.4 (32.5–34.3)
Rural	449 064 (71.1)	66.6 (65.7–67.5)
**Geographical region**		
North	129 312 (20.5)	13.8 (13.3–14.3)
Central	172 211 (27.2)	24.8 (24.1–25.5)
East	112 689 (17.8)	22.1 (21.4–22.9)
Northeast	87 228 (13.8)	3.5 (3.3–3.6)
West	51 687 (8.2)	14.2 (13.5–15.0)
South	78 749 (12.5)	21.6 (20.8–22.4)
**Covered by health insurance/health scheme**		
Yes	159 644 (25.3)	27.5 (27.0–28.0)
No	468 681 (74.2)	72.0 (71.5–72.4)
Don’t know	3551 (0.6)	0.5 (0.4–0.6)
**General healthcare seeking behaviour**		
Public	323 839 (51.3)	43.9 (43.4–44.4)
Private	287 727 (45.5)	52.3 (51.9–52.8)
Others^b^	20 310 (3.2)	3.7 (3.5–3.9)

^a^Includes Sikh, Buddhist, Jain, Jewish, Parsi/Zoroastrian, no religion and others.

^b^Home treatment, medical shop.

^c^Scheduled castes & tribes—disadvantaged caste and tribal population.

**Fig. 1:**
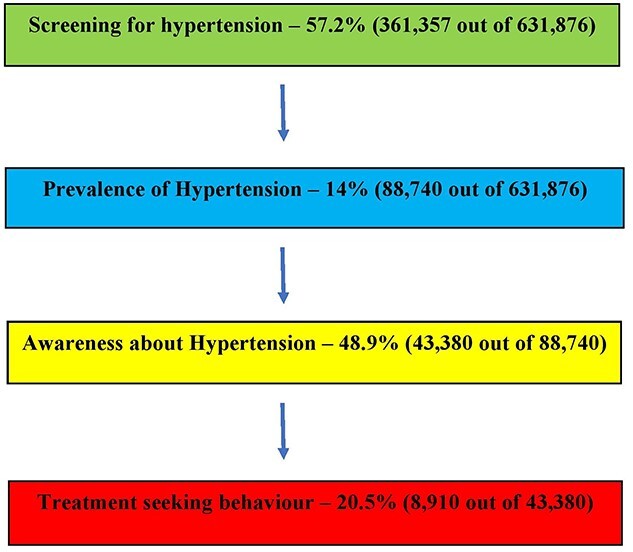
Hypertension care cascade pathway among adolescents and younger adults (15–39 years) in NFHS 4, India.

Hypertension care cascade among adolescents and younger adults are provided in Fig. 1. We found that 57.8% (95% CI: 57.4–58.1%) were screened for hypertension previously. The level of screening was better with higher age groups as only 27.9% adolescents were screened, whereas 64% those belonging to 20–29 years and 69.4% of those belonging to 30–39 years were screened. In total, 13.8% (95% CI: 13.6–14.1%) of the adolescents and younger adults had hypertension (self-reported/newly diagnosed). The burden of hypertension was also higher with each increasing age group with only 5.5% of adolescents had hypertension, whereas nearly 21% of those belonging to 30–39 years had hypertension. Amongst these young hypertensives, 51.1% (95% CI: 50.2–52.1%) of the participants were aware of their hypertension status. The level of awareness was better among those participants belonging to 20–29 years age group (57%) when compared with those belonging to 15–19 years (46.9%) or 30–39 years (47.5%). Amongst these self-reported participants, only 19.5% (95% CI: 18.7–20.3%) sought treatment and currently on medications (Table 2).

**Table 2:** Screening, prevalence, awareness and treatment-seeking behaviour for hypertension among adolescents and younger adults in India (stratified by age groups)

Characteristics	Frequency (unweighted proportion %)	Weighted proportion (95% CI)
**Screening for hypertension**		
**Screened for hypertension previously *n* = 631 876**	**361 357 (57.2)**	**57.8 (57.4–58.1)**
15–19 years	39 473 (27.4)	27.9 (27.5–28.4)
20–29 years	172 071 (63.3)	64.0 (63.6–64.5)
30–39 years	149 813 (69.5)	69.4 (68.9–69.9)
**Prevalence of young hypertension**		
**Overall prevalence of hypertension (self-reported and newly diagnosed cases) *n* = 631 876**	**88 740 (14.0)**	**13.8 (13.6–14.1)**
15–19 years	8357 (5.8)	5.5 (5.3–5.7)
20–29 years	34 132 (12.5)	12.6 (12.3–12.9)
30–39 years	46 251 (21.4)	20.9 (20.6–21.3)
**Awareness about hypertension**		
**Self-reported prevalence (awareness) of hypertension *n* = 88 740**	**43 380 (48.9)**	**51.1 (50.2–52.1)**
15–19 years	3586 (42.9)	46.9 (45.1–48.7)
20–29 years	18 285 (53.6)	57.0 (55.8–58.1)
30–39 years	21 509 (46.5)	47.5 (46.5–48.5)
**Treatment-seeking behaviour for hypertension**		
**Treatment-seeking behaviour (sought treatment & currently on medication) *n* = 43 380**	**8910 (20.5)**	**19.5 (18.7–20.3)**
15–19 years	647 (18.1)	17.2 (15.5–19.1)
20–29 years	2935 (16.1)	14.8 (13.9–15.7)
30–39 years	5328 (24.8)	24.2 (23.2–25.2)

Bold values signifies the statistically significant p-value

Table 3 shows the determinants of treatment-seeking behaviour among young hypertensives in India. All the variables from the univariable model were included in the multivariable model as they had *P* value < 0.20. In the adjusted analysis, age, gender, religion, residence, education, caste/tribe, geographical region and general healthcare seeking behaviour were found to be significant determinants of treatment-seeking behaviour.

**Table 3:** Determinants of treatment-seeking behaviour among the young hypertensives in India (*N* = 43 380)

Characteristics	Total	Sought treatment (weighted %)	Unadjusted prevalence ratio (95% CI)	*P* value	Adjusted prevalence ratio (95% CI)	*P* value
**Age category (in years)**						
15–19	3586	17.2	0.71 (0.64–0.79)	**<0.001**	0.70 (0.61–0.80)	**<0.001**
20–29	18 285	14.8	0.61 (0.57–0.65)	**<0.001**	0.63 (0.59–0.67)	**<0.001**
30–39	21 509	24.2	Ref	**—**	Ref	**—**
**Gender**						
Male	3909	16.4	0.83 (0.74–0.93)	**0.001**	0.84 (0.75–0.95)	**0.005**
Female	39 471	19.8	Ref	**—**	Ref	**—**
**Marital status**						
Never married	6610	16.1	Ref	—	Ref	—
Currently married	35 586	20.0	1.24 (1.13–1.36)	**<0.001**	0.97 (0.86–1.08)	0.58
Widowed/separated/divorced	1182	22.6	1.40 (1.18–1.67)	**<0.001**	0.98 (0.81–1.19)	0.88
**Religion**						
Hindu	30 245	18.6	Ref	**—**	Ref	—
Muslim	7026	23.3	1.25 (1.15–1.36)	**<0.001**	1.14 (1.04–1.25)	**0.007**
Christian	3513	20.7	1.11 (0.93–1.33)	0.25	1.15 (0.95–1.39)	0.15
Others^a^	2596	23.2	1.25 (1.08–1.43)	**0.002**	1.04 (0.91–1.21)	0.54
**Residence**						
Urban	14 883	18.1	0.88 (0.81–0.96)	**0.006**	0.87 (0.79–0.96)	**0.005**
Rural	28 497	20.4	Ref	**—**	Ref	—
**Education status (*N* = 43 279)**						
No formal education	7922	23.7	Ref	**—**	Ref	—
Primary	5440	22.2	0.94 (0.85–1.03)	0.17	0.99 (0.90–1.09)	0.91
Secondary	23 173	18.4	0.78 (0.72–0.83)	**<0.001**	0.88 (0.81–0.96)	**0.003**
Higher	6744	16.6	0.70 (0.63–0.79)	**<0.001**	0.86 (0.76–0.97)	**0.02**
**Caste/tribe (*N* = 41 095)**						
Scheduled caste	7997	18.3	0.80 (0.72–0.89)	**<0.001**	0.92 (0.83–1.02)	0.11
Scheduled tribe	6676	22.2	0.97 (0.87–1.09)	0.64	0.95 (0.84–1.07)	0.41
Other backward class	17 173	17.3	0.75 (0.70–0.82)	**<0.001**	0.89 (0.82–0.96)	**0.005**
None of the above	9249	22.9	Ref	**—**	Ref	—
**Wealth index**						
Poorest (I quintile)	5011	23.1	Ref	**—**	Ref	—
Poorer (II quintile)	7760	21.1	0.91 (0.83–1.00)	0.06	0.99 (0.90–1.10)	0.92
Middle (III quintile)	9688	17.8	0.77 (0.69–0.85)	**<0.001**	0.91 (0.82–1.02)	0.10
Richer (IV quintile)	10 720	18.6	0.81 (0.72–0.89)	**<0.001**	0.99 (0.89–1.12)	0.96
Richest (V quintile)	10 201	19.3	0.83 (0.75–0.93)	**0.001**	1.05 (0.91–1.20)	0.52
**Geographical region**						
North	11 009	20.5	0.73 (0.66–0.82)	**<0.001**	0.79 (0.69–0.91)	**0.001**
Central	7691	19.0	0.68 (0.62–0.76)	**<0.001**	0.76 (0.67–0.86)	**<0.001**
East	6995	24.2	0.87 (0.78–0.97)	**0.01**	0.94 (0.83–1.06)	0.34
Northeast	6669	27.9	Ref	**—**	Ref	—
West	2094	25.5	0.91 (0.79–1.06)	0.25	1.02 (0.86–1.20)	0.81
South	8922	14.7	0.53 (0.47–0.59)	**<0.001**	0.65 (0.57–0.75)	**<0.001**
**Covered by health insurance/health scheme (*n* = 43 139)**						
Yes	12 548	16.7	0.79 (0.74–0.86)	**<0.001**		0.05
No	30 591	21.0	Ref	**—**	Ref	—
**General healthcare seeking behaviour**						
Public	23 188	19.0	0.94 (0.87–1.00)	0.07	0.98 (0.91–1.05)	0.54
Private	19 296	20.2	Ref	**—**	Ref	—
Others^b^	896	14.3	0.71 (0.57–0.88)	**0.002**	0.63 (0.51–0.79)	**<0.001**

^a^Includes Sikh, Buddhist, Jain, Jewish, Parsi/Zoroastrian, no religion and others.

^b^Home treatment, medical shop, others.

Bold values signifies the statistically significant p-value

Participants belonging to 15–19 years age group (aPR = 0.70; 95%CI: 0.61–0.80) and 20–29 years age group (aPR = 0.63; 95%CI: 0.59–0.67) had lesser treatment-seeking behaviour when compared to the participants belonging to higher age group (30–39 years). Males had lesser treatment-seeking behaviour for hypertension (aPR = 0.84; 95%CI: 0.75–0.95) when compared to females. Muslims had significantly better treatment-seeking behaviour for hypertension when compared to Hindus (aPR = 1.14; 95%CI: 1.04–1.25). A smaller number of participants in urban areas had sought treatment for hypertension (aPR = 0.87; 95%CI: 0.79–0.96) compared with people in rural residence. Participants having secondary (aPR = 0.88; 95%CI: 0.81–0.96) and higher educational qualification (aPR = 0.86; 95%CI: 0.76–0.97) had significantly lower treatment-seeking behaviour when compared to participants with no formal education. Participants belonging to Northern (aPR = 0.79; 95%CI: 0.69–0.91), Central (aPR = 0.76; 95%CI: 0.67–0.86), Southern region (aPR = 0.65; 95%CI: 0.57–0.75) had significantly lower treatment-seeking behaviour when compared to participants in the Northeast region. Participants who prefer home treatment, medical shop or other form of care for seeking general healthcare (aPR = 0.63; 95%CI: 0.51–0.79) had significantly lower treatment-seeking behaviour compared to those who seek general healthcare from private health facilities.

## Discussion

### Main findings of the study

In the current study, the burden of young hypertension in India was 13.8%. We have found that 42.2% of the young adults were never screened for hypertension before. Among the young hypertensives, only 51.1% knew about their status and only 19.5% of them sought treatment. We also found that the adolescents, males, Hindus, urban population, higher education and residing in Northern, Central and Southern region had poor treatment-seeking behaviour.

### What is already known on this topic

The overall prevalence of young hypertension in India was found to be 13.8% (95% CI: 13.6–14.1%). Similar finding was found in the large prospective SITE study (Screening India’s twin epidemic) where 12.7% of the individuals below 40 years had hypertension.^6^ Large-scale South Asian study conducted by Prasad *et al*. showed that 11.9% of young adults had hypertension.^18^ Other small-scale studies around India showed that the prevalence of young hypertension ranging from 10 to 17%.^19–21^ There is an increasing burden of young hypertension over the past decade in India because of adopting unhealthy lifestyle changes like unhealthy dietary habits, physical inactivity and tobacco and alcohol use at an earlier age. A retrospective analysis among young hypertensives showed that ~30% of hypertension among younger adults was due to secondary causes.^22^

### What this study adds

In the current study, we have found that 42.2% of the young adults were never screened for hypertension before. Among the young hypertensives diagnosed in the study, only 51.1% knew about their hypertension status and only 19.5% of them sought treatment and currently on medications. Existing guidelines on hypertension in India has provided comprehensive guide on screening, diagnosis, assessment and management of hypertension.^15^ In addition, introduction of newer programmes for population level screening of hypertension and awareness campaigns are being conducted.^15^^,^^16^ Almost half of the younger adults were never screened for hypertension or unaware of their hypertensive status and ~80% of young hypertensives did not seek treatment. This shows that our country is slow or lagging behind in the secondary prevention of hypertension, which involves early diagnosis by screening and adequate treatment of the condition. Hence, the combination of active mode of population level and high-risk group screening and the passive mode of opportunistic screening of all the younger adults for hypertension will help to improve the scenario. Dropout from seeking care should be avoided by following up the patient through frontline workers like ASHA, ANM or AWW during their regular home visits. Once diagnosed, primary health facility nearest to the patient’s residence need to be informed in carrying out the above-mentioned activities.

We also identified certain determinants of poor treatment-seeking behaviour for hypertension, which will help devise targeted interventions for early initiation of treatment and increase the coverage among younger hypertensives. We found that participant belonging to 15–19 years and 20–29 years had poor treatment-seeking behaviour compared with 30–39-year-old participants with hypertension. Possible reasons for such finding could be the desire to handle their own problems, lower perceived susceptibility and perceived need for seeking help and financial concerns.^23^ However, further qualitative exploration is required to understand and address their concerns in seeking treatment for hypertension. We also found that males had poor treatment-seeking behaviour for hypertension compared to females. Males tend to have poor health-seeking behaviour in general irrespective of the condition.^24^^,^^25^ It has also been reported that the men only seek care only during emergencies or in the later stages of chronic illnesses.^26^^,^^27^ Hence, males remain an important high-risk group that need to be targeted for improving their care-seeking behaviour especially for chronic conditions like hypertension.

We also found that the urban population, participants with higher educational qualifications and people belonging to the Southern region had poor treatment-seeking behaviour for hypertension. Some of these findings are surprising given the higher awareness about the disease condition and its complications amongst people with higher educational qualifications. In urban area, irrespective of the higher accessibility to healthcare facilities, there is predominance of the private sector making it difficult for the urban poor to access and afford the care. Hence, concerted efforts should be made to strengthen the public health system, especially the primary healthcare facilities and door-step services for the needy.^28^^,^^29^ Though, the Southern region contains some of the best-performing states in terms of almost all the health indicators in the country, the younger population was found to have poor treatment-seeking behaviour for hypertension in our study. Hence, it is important to explore the reasons behind such finding through qualitative survey.

A major strength of the study is analysing data from the nationally representative survey with a higher response rate to determine the estimate of hypertension cascade of care amongst young hypertensives. This increases the generalisability of the results as the sample was larger and representative of the younger population in India. The current study contributes to the limited evidence available regarding the treatment-seeking behaviour among the younger hypertensives.

Current study also has certain implications for public health practice. The current study provides valuable insights into the treatment-seeking behaviour among young hypertensives in India. It emphasizes the importance of identifying the adolescents and younger adults at high-risk of dropping out from the treatment. As most health promotion and screening strategies are targeted towards the middle-aged and elderly population, there is a need to develop newer strategies or adopt successful strategies from other countries to reduce the dropout and improve the treatment initiation among younger adults. Life course approach is one such strategy, which was proven to be effective in preventing the development of NCDs and emphasizes about the importance of early diagnosis and initiation of treatment. Interventions implemented at schools, colleges and workplaces are important to reduce the dropout rate, targeting adolescents and younger adults. However, further interventional study needs to be done to help devise newer interventions for improving their treatment-seeking behaviour.

### Limitations of the study

The study's limitations were the cross-sectional design, which makes it difficult to infer the causal relationship. The data on diagnosis of young hypertension and their treatment-seeking behaviour were collected during the household survey without cross-checking the details from the health facility. Hence, there can be an underestimation of the known case of hypertension or their treatment-seeking behaviour. We also could not explore the role of certain important factors like perceived susceptibility, perceived severity across the selected age groups in poor treatment-seeking behaviour, perceived need for seeking help, etc., because of data limitations.^23^

## Conclusion

The current study found that more than 1 in 10 younger adults in India have hypertension. Only half of these hypertensive participants were aware of their hypertension status and only one-fifth of these sought treatments and were currently on medication. Adolescents, males, Hindus, urban population, participants with higher educational qualification and belonging to Northern, Central and Southern region had poor treatment-seeking behaviour for hypertension. Hence, a holistic approach to screen all adolescents and younger adults should be made for early diagnosis and early initiation of treatment to prevent the development of complications in the future.

## Notes

**Yuvaraj Krishnamoorthy** Assistant Professor

**Sathish Rajaa** Assistant Professor

**Sudheera Sulgante** Assistant Professor

**Palanivel Chinnakali** Assistant Professor

**Nidhi Jaswal** Technical Coordinator (Hypertension Program)

**Sonu Goel** Professor, Adjunct Clinical Associate Professor
